# Bismuth Subcarbonate Decorated Reduced Graphene Oxide Nanocomposite for the Sensitive Stripping Voltammetry Analysis of Pb(II) and Cd(II) in Water

**DOI:** 10.3390/s20216085

**Published:** 2020-10-26

**Authors:** Guo Zhao, Mohammed Sedki, Shengcun Ma, Claudia Villarreal, Ashok Mulchandani, David Jassby

**Affiliations:** 1 College of Artificial Intelligence, Nanjing Agricultural University, Nanjing 210031, China; zhaoguo@njau.edu.cn; 2 Department of Chemical and Environmental Engineering, University of California, Riverside, CA 92521, USA; 3 Materials Science and Engineering Program, University of California, Riverside, CA 92521, USA; mabue002@ucr.edu (M.S.); cchav021@ucr.edu (C.V.); 4 Department of Civil and Environmental Engineering, University of California, Los Angeles, CA 90095, USA; shengcun@ucla.edu (S.M.); jassby@ucla.edu (D.J.); 5 Materials Science and Engineering, Instituto Tecnológico de Costa Rica, 30101 Cartago, Costa Rica; 6 Center for Environmental Research and Technology (CE-CERT), University of California, Riverside, CA 92507, USA

**Keywords:** bismuth subcarbonate, square-wave anodic stripping voltammetry, reduced graphene oxide, heavy metals detection

## Abstract

In this paper, bismuth subcarbonate (BiO)_2_CO_3_-reduced graphene oxide nanocomposite incorporated in Nafion matrix ((BiO)_2_CO_3_-rGO-Nafion) was synthesized and further applied, for the first time, in the sensitive detection of Pb(II) and Cd(II) by square-wave anodic stripping voltammetry (SWASV). The as-synthesized nanocomposites were characterized by energy-dispersive spectroscopy (EDS), Raman spectroscopy, scanning electron microscopy (SEM), Fourier transform infrared spectroscopy (FTIR), X-ray diffraction (XRD), cyclic voltammetry (CV), and electrochemical impedance spectroscopy (EIS). (BiO)_2_CO_3_ composite plays a key role in the improvement of the detection sensitivity, which can form multicomponent alloy with cadmium and lead. Additionally, the unique structure of rGO can enlarge the surface area and provide abundant active sites. Moreover, Nafion incorporation in the nanocomposite can effectively increase the adhesion and mechanical strength of the film, and further improve the preconcetration ability due to the cation-exchange capacity of its abundant sulfonate groups. As expected, the (BiO)_2_CO_3_-rGO/Nafion nanocomposite-modified glassy carbon electrode ((BiO)_2_CO_3_-rGO-Nafion/GCE) achieved low detection limits of 0.24 μg/L for Pb(II) and 0.16 μg/L for Cd(II), in the linear range of 1.0–60 μg/L, and showed some excellent performance, such as high stability, good selectivity, and sensitivity. Finally, synthetic water samples were prepared and further used to verify the practicability of the (BiO)_2_CO_3_-rGO-Nafion/GCE with satisfactory results.

## 1. Introduction

As two of the most toxic heavy metals (HMs), cadmium and lead have been regarded as two serious carcinogens. With the urban construction and industry development, elevated concentrations of toxic elements can be found in surface water and ground water because of the manufacturing of pigments, circuit boards, and batteries; the application of phosphate fertilizers; and the combustion of fossil fuels [1], which can potentially harm the health of living organisms [2]. Additionally, as traditional water supplies for thermoelectric power plants are coming under increased stress from a growing population, rises in temperature, and stricter environmental regulations, there is an increased interest in exploiting marginal water sources, such as municipal wastewater [3,4,5]. Municipal wastewater, in particular, is of interest because of its widespread availability and relatively consistent quality [4]. However, the complex nature of municipal wastewater, which can increase corrosion in the system, as well as the high metal contaminant loads found in certain wastewater streams, make their use in thermoelectric power plants challenging [6]. An accurate heavy metal detection method that can operate autonomously and accurately will enable the safer use of these marginal waters for cooling and other power plant-related activities, and also prevent the heavy metals from harming the health of human beings.

So far, many techniques have been introduced to carry out the reliable and accurate analysis for the lead and cadmium based on spectroanalysis, such as inductively coupled plasma–mass spectrometry [7], atomic fluorescence spectrometry [8,9], inductively coupled plasma–optical emission spectrometry [10], and atomic absorbance spectrometry [11]. Nonetheless, these spectroscopic methods are considered more time-consuming and expensive than electrochemical methods, and it is difficult to use them for onsite analysis [12,13]. Because of the advantages of anodic stripping voltammetry (ASV), such as instrument portability, low cost, and fast response, it has been regarded as one of the most promising HMs detection technologies. Usually, the stripping voltammetry measurement is carried out using a three-electrode system, i.e., reference electrode (RE), counter electrode (CE), and working electrode (WE). The WEs play a key role in the detection sensitivity of this method. In the past few decades, bismuth-based materials modified electrodes have gradually become a successful alternative to the mercury electrodes due to their interesting characteristics, such as low toxicity, a wide electrochemical potential window, and the ability to form alloys with heavy metals [14,15], which helps detect them at trace levels [16,17,18,19,20].

According to the substrate of bismuth-based materials for the modification of electrodes, there are three general ways to fabricate a Bi electrode [21,22]. (1) Ex-site method: First, the electrode is immersed into a Bi(III) solution applied with a negative potential to reduce the Bi(III) into metallic Bi. After that, the Bi film is electroplated on surface of the electrode, i.e., the ex-site Bi modified electrode. (2) In situ method: Bi(III) ions are added to buffer solution before the measurement, the target HMs and Bi(III) ions are electrodeposited to the electrode at the same time using a negative potential. (3) “Bulk” method: Bi is used for the modification of electrode during the electrode production as a bismuth precursor and then reduced to metallic Bi with a reduction potential [23].

As one of the electrode modification methods, in situ modification of glassy carbon electrodes (GCEs) has the advantages of good sensitivity and repeatability, which enable its wide application in the fabrication of electrochemical sensors. Kefala et al. [24] developed an electrochemical sensor based on a polymer-coated bismuth film electrode for the determination of trace metals by sequential-injection analysis. Nevertheless, to keep the concentration of Bi(III) constant in the testing solution, a closed liquid condition is essential for the application of Bi(III) in situ modified electrodes, which leads to the consumption of bismuth solution and may lead to the secondary pollution. As compared with in situ modification method, the sensitivity of ex-situ modified electrodes needs to be further improved [25,26]. In addition, the reusability of Bi(III) ex situ modified electrode is not as good as Bi(III) in situ modified electrode due to the potential loss of metallic Bi electroplated on the electrode surface during the cleaning step and stripping step. However, few papers reported how to prevent the loss of Bi-based materials during the measurement of stripping voltammetry. Another alternative method is to directly modify the material onto the electrode surface to form a thick Bi film working electrode. Compared with the traditional bismuth modification methods, this method is more convenient to use, because it requires neither the Bi(III) plating solution nor the conductive electrode substrate.

In the present work, we introduced bismuth subcarbonate-reduced graphene oxide nanocomposite incorporated in Nafion matrix ((BiO)_2_CO_3_-rGO-Nafion) for the direct modification of the glassy carbon electrode. Our proposed method herein depends on the fact that the oxygen-containing groups on the GO nanosheets should chelate the Bi^3+^. Hence, they act as the nuclei for the Bi NPs on the nanosheets during the reduction process. The characterizations of the prepared hybrid indicated that the bismuth was in the form of bismuth subcarbonate, and it is worth mentioning that this is the first time that this nanocomposite was used in the electrochemical detection of cadmium and lead. More details are revealed in the Results and Discussion sections. The proposed (BiO)_2_CO_3_-rGO-Nafion-modified glassy carbon electrode ((BiO)_2_CO_3_-rGO-Nafion/GCE) exhibited a comparable or even higher sensitivity than some in situ and/or ex situ bismuth film-modified electrodes. The combination of (BiO)_2_CO_3_, rGO, and Nafion resulted in many intriguing synergistic effects. In particular, rGO nanosheets increased the electrical conductivity, as well as the surface area of the composite, while (BiO)_2_CO_3_ played the role of adsorbing heavy metals for preconcentrating them on electrode surface to help detect lower concentrations. In addition, Nafion helped improve the mechanical stability of the composite at electrode surface and helped improve the heavy metals absorption by cation-exchange. We also reported the synthesis method and the characterization of the proposed (BiO)_2_CO_3_-rGO-Nafion nanocomposite using different techniques (Fourier transform infrared spectroscopy (FTIR), X-ray diffraction (XRD), Raman spectroscopy, scanning electron microscopy (SEM), energy-dispersive spectroscopy (EDS), cyclic voltammetry (CV), and electrochemical impedance spectroscopy (EIS)) and the analytical application of (BiO)_2_CO_3_-rGO-Nafion/GCE for the determination of Pb(II) and Cd(II) in the synthetic water samples.

## 2. Experimental

### 2.1. Materials

Graphite powder, bismuth nitrate (Bi(NO_3_)_3_·5H_2_O), N, N-dimethylformamide (DMF), ethylene glycol (EG), sodium acetate trihydrate, sulfuric acid (H_2_SO_4_, 98%), phosphoric acid (H_3_PO_4_, 85%), potassium permanganates (KMnO_4_), hydrogen peroxide (H_2_O_2_, 30%), and hydrochloric acid (HCl, 37%) were purchased from Fisher Scientific (Chino, CA, USA). Nafion (1 wt% in ethanol) and Pb(II) and Cd(II) solutions (1 mg/mL) were obtained from Sigma Aldrich (St. Louis, MO, USA). All solutions were prepared with Milli-Q water (Riverside, CA, USA).

### 2.2. Instruments

Electrochemical analysis, i.e., CV, EIS, and square-wave anodic stripping voltammetry (SWASV) were performed using a 6005E electrochemical workstation (CH Instruments Inc., Austin, TX, USA). All experiments were carried out in a three-electrode (Pt wire counter electrode, Ag/AgCl reference electrode, glassy carbon working electrode (GCE)) electrochemical cell with 20 mL volume. A magnetic stirrer was used to stir the test solution during the deposition step. FTIR spectroscopy was performed using a Nicolet 6700 FT-IR spectrophotometer (Thermo Fisher Scientific, Waltham, MA, USA). Raman spectra was collected on a Horiba LabRAM monochromator (Horiba, Irvine, CA, USA) equipped with a 532 nm (2.33 eV) laser, using a 100× objective (NA = 0.94) at room temperature. XRD measurements were taken at room temperature with the Empyrean diffractometer from Malvern Panalytical (Westborough, MA, USA) on glass slides at room temperature in the 2θ range of 10° to 70°. SEM and energy-dispersive X-ray spectroscopy (EDX) were performed using FEI NovaNanoSEM 450 (Thermo Fisher Scientific, Waltham, MA, USA).

### 2.3. Preparation of Graphene Oxide (GO) Nanosheets

GO nanosheets were prepared using the Improved Hummers Method (IHM) [27], with some fine changes in reaction temperature and time (3 h instead of 12 h), as reported previously [28]. Briefly, 1 g of graphite powder was added to a mixture of 120 mL of 98% H_2_SO_4_ and 13 mL of 98% H_3_PO_4_ and stirred magnetically for 30 min at 15 °C for homogenization. Then, 6 g of potassium permanganate was added to the homogenized mixture portion-wise and the reaction temperature was elevated to 45 °C for 3 h with magnetic stirring. The reaction was quenched by pouring the reaction mixture, very slowly and carefully, into a mixture of 150 mL of deionized water and 20 mL of hydrogen peroxide. The reaction mixture was kept static overnight to settle down the prepared GO nanosheets for easy decanting of the acids and byproducts. The prepared graphite oxide sheets were washed several times by centrifugation with hydrochloric acid and deionized water until the pH of the suspended GO was close to 6.0, and were further exfoliated by bath sonication for 10 min to form GO nanosheets, which were separated by centrifugation and dried at 50 °C.

### 2.4. Synthesis of (BiO)_2_CO_3_-rGO Nanocomposite

First, 100 mg of bismuth nitrate (Bi(NO_3_)_3_ 5H_2_O) were dissolved in 20 mL of GO suspension (5 mg/mL) in a mixture of 1:1 water and ethylene glycol. The mixture was stirred for 30 min at 60 °C. Then, 0.51 g of sodium borohydride was added portionwise to the Bi^3+^-GO suspension in water/ethylene glycol. The reduction reaction proceeded for 2 h at 60 °C under magnetic stirring. The prepared (BiO)_2_CO_3_-rGO hybrid nanostructure was then washed a few times with deionized water by centrifugation and dried in an oven at 50 °C.

### 2.5. Electrode Fabrication and Electrochemical Detection of Cd(II) and Pb(II)

Before modification with the (BiO)_2_CO_3_-rGO nanocomposite on its surface, the GCE (3 mm diameter) was polished with 0.05 μm alumina powder on a microcloth pad and washed ultrasonically in 1:1 HNO_3_ and water followed by absolute ethanol and finally water. Then, 1 mg of (BiO)_2_CO_3_-rGO composite was added to 4 mL DMF, followed by 800 μL of a 0.5 wt% of Nafion solution (in ethanol), and sonicated until the (BiO)_2_CO_3_-rGO was dispersed uniformly. Next, 8 μL of this suspension was drop-casted on the surface of polished GCE and dried in an oven at 60 °C. For comparison, other electrodes were also prepared similarly.

The electrochemical measurements of SWASV were performed in a 0.2 M acetate buffer solution (pH 5.0) containing different concentrations of target HMs, i.e., Cd(II) and Pb(II). Unless stated otherwise, HMs were pre-deposited on the working electrode at −1.2 V vs. Ag/AgCl for 120 s under stirring. At this point, the stirring was stopped, the solution was equilibrated for 10 s, and an anodic stripping voltammogram was obtained from −1.1 to −0.3 V with the potential step, amplitude, and frequency of 5 mV, 25 mV, and 25 Hz, respectively. The composition of the internal solution of Ag/AgCl reference electrode was saturated KCl with a concentration of 3 M. For the analysis of HMs in synthetic Yamuna River water, 9 mL of the simulated water sample was mixed with 1 mL of 2 M acetate buffer (pH 5.0) to adjust the pH to 5.0 with 0.2 M acetate followed by deposition and SWASV. Electrochemical impedance spectroscopy (EIS) measurements were collected from 100 kHz to 0.1 Hz with an amplitude of 5 mV using [Fe(CN)_6_]^3−/4−^ as a redox probe.

### 2.6. Preparation of Synthetic Yamuna River Water

Water simulating the chemical composition of the Yamuna River in New Delhi, India, was used to evaluate the performance of the proposed sensor for the environmental water source. The simulated water was prepared by dissolving the following chemicals in 1 L of deionized water: 150 mg magnesium nitrate, 60 mg ammonium chloride, 500 mg potassium chloride, 50 mg sodium citrate, and 500 mg calcium chloride. The concentration of the total dissolved solids (TDS) was 1260 ppm.

## 3. Results and Discussion

### 3.1. Characterization of (BiO)_2_CO_3_-rGO Nanocomposite 

#### 3.1.1. XRD Analysis

**Figure 1:**
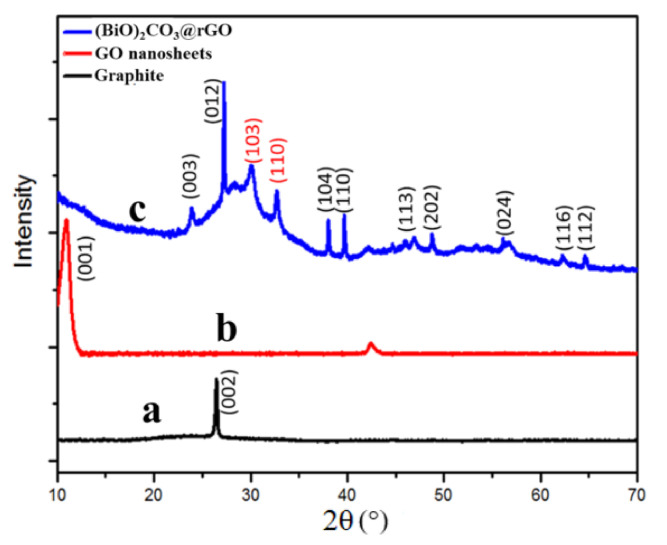
X-ray diffraction (XRD) analysis of (**a**) graphite, (**b**) graphene oxide, and (**c**) (BiO)_2_CO_3_-rGO nanosheets. The samples were prepared as thin films on glass slides, by drop-casting and room-temperature drying.

The crystallographic structures of the as-prepared GO and (BiO)_2_CO_3_-rGO nanomaterials were studied using XRD analysis (Figure 1). The interplanar distance between graphene sheets in the hexagonal structure of graphite was *d* = 0.345 nm, corresponding to 2θ = 26° (Figure 1, trace a) [29], while the oxidation process pushed the layers away and increased the interplanar distance to *d* = 0.743 nm, which corresponds to 2θ = 10.6° (Figure 1, trace b). Regarding (BiO)_2_CO_3_-rGO (Figure 1, trace c), the XRD peaks matched the diffraction peaks of the rhombohedral-phased bismuth (JCPDS card no. 05-0519). However, the two peaks at 30.02° and 32.66° did not match the bismuth pattern. They matched the (103) and (110) planes of the diffractogram of bismuth subcarbonate ((BiO)_2_CO_3_) (National Bureau of Standards, USA), and the starting materials of GO and ethylene glycol could have been the carbon sources for the formation of the subcarbonate [30]. Hence, the prepared nanocomposite was (BiO)_2_CO_3_-rGO.

#### 3.1.2. FTIR Spectroscopic Analysis

**Figure 2:**
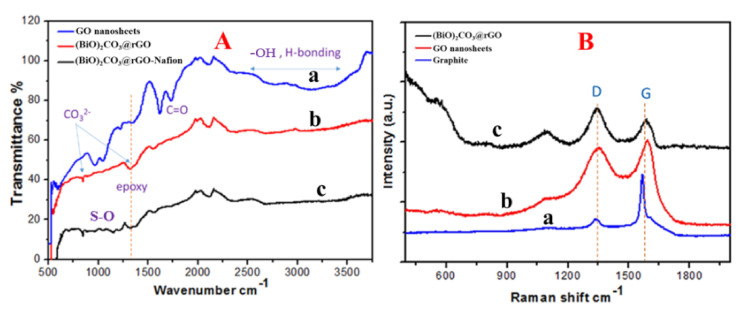
(**A**) Fourier transform infrared spectroscopy (FTIR) spectra of graphene oxide (GO) nanosheets (**a**), (BiO)_2_CO_3_-rGO nanocomposite (**b**) and (BiO)_2_CO_3_-rGO-Nafion nanocomposite (**c**); (**B**) Raman spectra recorded at the 514 nm laser beam of graphite (**a**), GO nanosheets (**b**), and (BiO)_2_CO_3_-rGO nanocomposite (**c**).

The FTIR spectrum of GO nanosheets synthesized by the modified IHM, as previously reported by Sedki et al. [28], (Figure 2A, trace a), exhibited peaks of the following groups: O–H stretching vibration at 3420 cm^−1^, overlapping with the broad peak of O–H from carboxyl groups from 2500 to 3500 cm^−1^, C=O stretching vibrations at 1750 cm^−1^, C=C from unoxidized sp^2^ hybridized carbon atoms at 1620 cm^−1^, and C–C vibrations at 1250 cm^−1^, in agreement with literature [27]. It is hypothesized that, in IHM, the hydroxyl, carbonyl, and epoxy groups are formed on the basal plane and help push graphite layers apart to facilitate exfoliation of graphene oxide nanosheets during exfoliation, whereas the carboxyl groups are formed through breaking some terminal rings. In addition, the recorded FTIR of the in situ synthesized (BiO)_2_CO_3_-rGO nanocomposite (Figure 2A, trace b) showed the disappearance of the broad peak of O–H groups at 2500–3500 cm^−1^, confirming the reduction process [31]. The introduction of two new peaks at 873 and 1350 cm^−1^ can be attributed to the vibrations of carbonate [32], which confirms the formation of bismuth subcarbonate. Moreover, the addition of Nafion to the nanocomposite (Figure 2A, trace c) was confirmed by the –SO_3_– symmetric stretching vibrations peaks at 1056 cm^−1^ and asymmetric vibrations of –SO_3_– groups at 1201 cm^−1^ [33].

#### 3.1.3. Raman Spectroscopic Analysis

The Raman spectroscopic analysis of GO and (BiO)_2_CO_3_-rGO nanocomposite can be seen in Figure 2B. Graphene materials showed two main peaks in Raman spectra. The first is the G-band that appeared around 1580 cm^−1^, and the second is the D-band that appeared around 1350 cm^−1^. The G-band is attributed to the in-phase vibrations of the sp^2^ lattice, while the D-band refers to the structural defects and disorders. The I_D_/I_G_ ratio was almost 0.1 in graphite (Figure 2B, trace a), 0.9 in GO (Figure 2B, trace b), and 1.2 in (BiO)_2_CO_3_-rGO (Figure 2B, trace c), which illustrates introducing lattice defects and decreasing the average size of the sp^2^ domains by the oxidation process in GO, and more defects by reduction of GO into rGO [34]. These results of rGO match the literature [35] and can be attributed to eliminating the defects and, at the same time, introducing a higher number of new smaller graphitic domains. Moreover, the Raman bands at 100, 512 and 1070 cm^−1^ are attributed to the vibrations of bismuth subcarbonate [36].

#### 3.1.4. SEM Imaging

**Figure 3:**
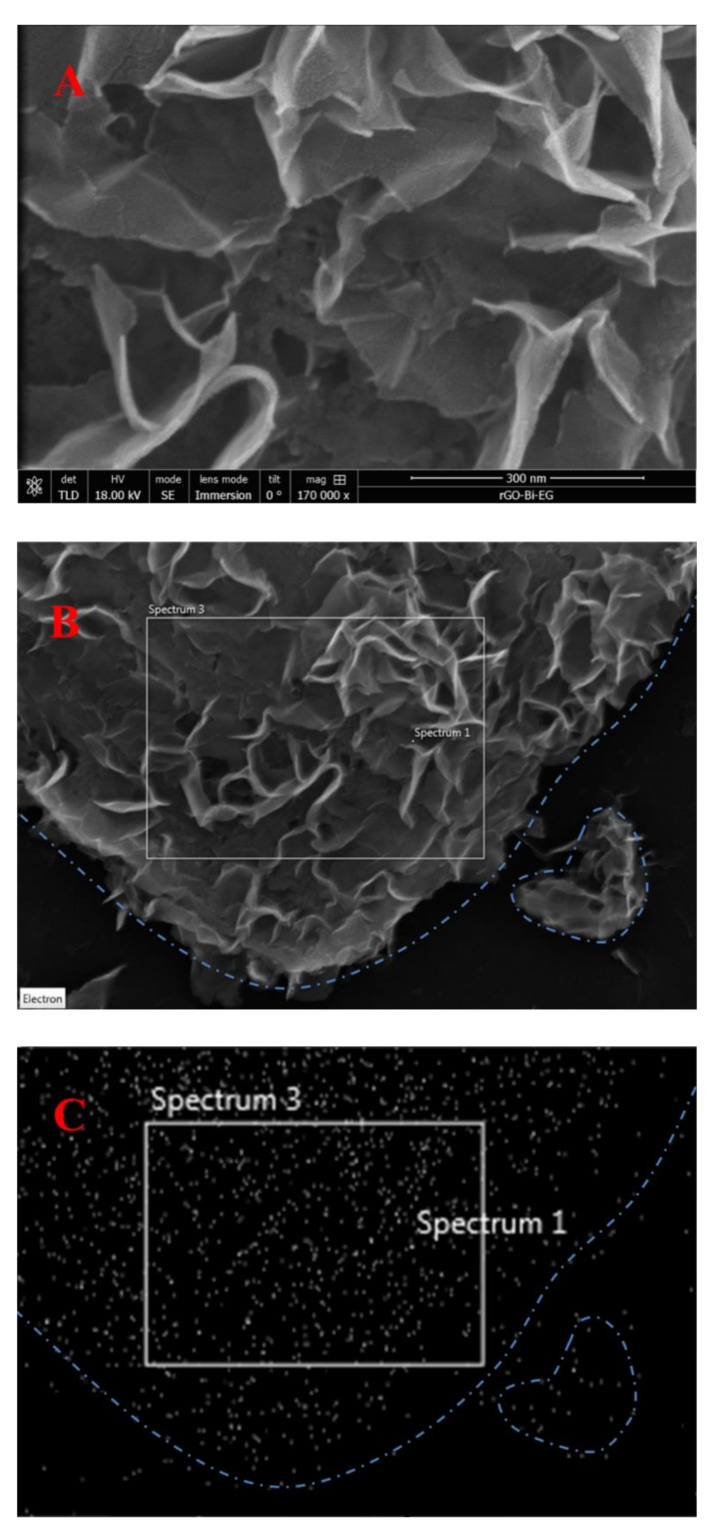
Scanning electron microscopy (SEM) of (BiO)_2_CO_3_-rGO (**A**) and energy-dispersive X-ray spectroscopy (EDX) mapping of Bi (**B**,**C**). The distribution density of Bi in micrograph (**B**) matches the distribution density of rGO nanosheets in micrograph (**C**).

Figure 3 shows SEM images and EDX mapping of the (BiO)_2_CO_3_-rGO nanocomposite prepared in this work. The SEM image (Figure 3A) demonstrates a uniformly distributed thin film of (BiO)_2_CO_3_ NPs, covering almost the full surface of the rGO nanosheets (Figure 3A). Furthermore, the EDX mapping confirmed that the (BiO)_2_CO_3_, or simply Bi NPs, were well distributed on rGO nanosheets, as the density of the NPs matched the density of the rGO nanosheets (Figure 3B,C). The ethylene glycol diluted by water was the viscous medium, which slowed the reduction rate of Bi^3+^ ions in the reaction and therefore controlled the size distribution and played the role of the surfactant that prevented nanoparticles agglomeration/aggregation [37,38].

### 3.2. Electrochemical Characteristics of Different Modified Electrodes

**Figure 4:**
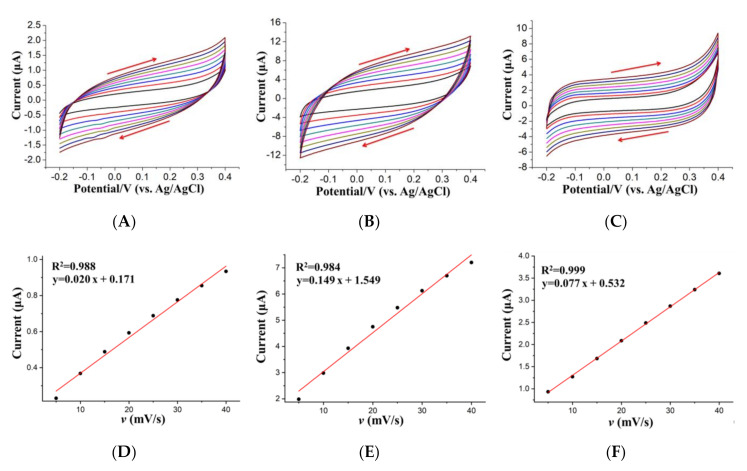
Cyclic voltammograms for the glassy carbon working electrode (GCE) (**A**), (BiO)_2_CO_3_-rGO/GCE (**B**), and (BiO)_2_CO_3_-rGO-Nafion/GCE (**C**), collected at different potential scanning rates (5, 10, 15, 20, 25, 30, 35 and 40 mV/s) in 1.0 M KOH, and the corresponding calibration curves of GCE (**D**), (BiO)_2_CO_3_-rGO/GCE (**E**), and (BiO)_2_CO_3_-rGO-Nafion/GCE (**F**), which are related to the charge transfer currents at the potential of 0.2 V and different potential scanning rates. The charge transfer currents were calculated by the equation of $\frac{I1-I2}{2}\;$ (I1 and I2 are the anodic and cathodic current density at 0.2 V, respectively [39]).

The surface area of the electrode plays a very important role in the sensitivity of HMs detection. Therefore, the surface area of GCE and the different modified electrodes, i.e., (BiO)_2_CO_3_-rGO/GCE and (BiO)_2_CO_3_-rGO-Nafion/GCE, were determined by cyclic voltammetry, as shown in Figure 4.

Figure 4 shows the cyclic voltammograms with different potential scanning rates (5 to 40 mV/s) obtained in 1.0 M KOH using GCE (Figure 4A), (BiO)_2_CO_3_-rGO/GCE (Figure 4B), and (BiO)_2_CO_3_-rGO-Nafion/GCE (Figure 4C). The slope of the linear calibration curves, which were built up by charge transfer current and potential scanning rate, was used to calculate the electrochemical active surface area of GCE (Figure 4D), (BiO)_2_CO_3_-rGO/GCE (Figure 4E), and (BiO)_2_CO_3_-rGO-Nafion/GCE (Figure 4F), respectively, and the main equations are summarized below [40,41,42].

(1) $$i\;=\;i_{c}+\;i_{f}$$

where i is the current flowing through the electrode, which consists of two parts, i.e., the charge transfer current acting on the double layer ($i_{c}$) and the faradic current acting on the redox reaction ($i_{f}$). Additionally, the charge transfer current $i_{c}$ also can be divided into two parts, as shown in Equation (2).

(2) $$i_{c}=C_{dl}\frac{d\varphi }{dt}+\varphi \frac{dC_{dl}}{dt}$$

where $C_{dl}$ and φ are the capacitance and potential of the double layer, respectively. One part of $i_{c}$ is from the charging and discharging of the double layer caused by the changes of electrode potential, i.e., $\frac{d\varphi }{dt}$, which changes the charged state of the electrode/solution interface. $\frac{dC_{dl}}{dt}$ is the other part of $i_{c}$, which is from the changes of $C_{dl}$. During the linear potential scanning, $i_{c}$ is not equal to 0, because the scanning rate is constant. However, when the potential scanning range is relatively small, the charged state of the double-layer interface can be approximately considered as unchanged, so the corresponding $C_{dl}$ is also constant, and so that $\frac{dC_{dl}}{dt}$ is equal to 0, which can be ignored. Therefore, $C_{dl}$ is equal to $\frac{i_{c}\cdot dt}{d\varphi }$, which is the slope of the equations shown in Figure 4D–F.

(3) Surface area = CdlCideal

Finally, the electrochemical active area of bare GCE, (BiO)_2_CO_3_-rGO/GCE, and (BiO)_2_CO_3_-rGO-Nafion/GCE were calculated to be 33.29, 248.02 and 128.17 mm^2^, respectively, based on Equation (3), where $C_{ideal}\;$(60 μF cm^-2^) is the double-layer capacitance of the oxide electrode with an ideal smooth surface [40,41,42]. The results show that specific surface area of the bare GCE was effectively increased by rGO, which provided more active sites for the attachment and growth of Bi NPs.

**Figure 5:**
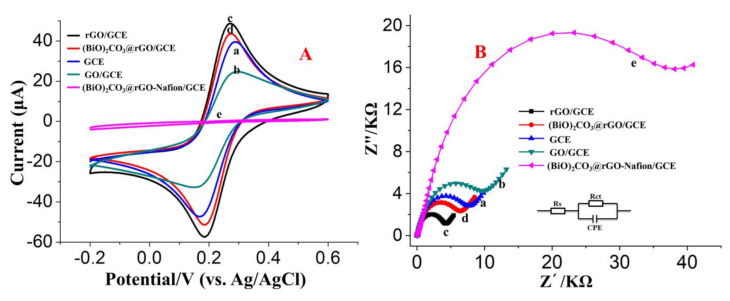
Cyclic voltammograms (**A**) and Nyquist plots of electrochemical impedance spectra (**B**) of 5 mM [Fe(CN)_6_] ^3−/4−^ in 0.1 M KCl: bare GCE (**a**), GO/GCE (**b**), rGO/GCE (**c**), (BiO)_2_CO_3_-rGO/GCE (**d**), and (BiO)_2_CO_3_-rGO-Nafion/GCE (**e**). The electrochemical impedance spectroscopy (EIS) was collected from 100 kHz to 0.1 Hz with an amplitude of 5 mV.

The electrochemical characteristics of different modified GCEs were investigated using CV measurements with the redox probe of [Fe(CN)_6_]^3−/4−^. The redox currents of [Fe(CN)_6_] ^3−/4−^ at the bare GCE (curve a), GO/GCE (curve b), rGO/GCE (curve c), (BiO)_2_CO_3_-rGO/GCE (curve d), and (BiO)_2_CO_3_-rGO-Nafion/GCE (curve e) are shown in Figure 5A. A pair of well-defined redox peaks was obtained on the bare GCE (curve a). GO/GCE showed lower redox currents than bare GCE, which can be attributed to the poor conductivity of GO and its negative charges. On the other hand, rGO/GCE exhibited more redox currents than GCE, due to the excellent electrical conductivity and the higher surface area of rGO. Compared to curve c, two decreased redox peaks can be found on (BiO)_2_CO_3_-rGO/GCE (curve d), which demonstrate that the (BiO)_2_CO_3_ has a poor electron transfer capacity [43]. Under the same conditions, the redox peaks of the (BiO)_2_CO_3_-rGO-Nafion/GCE (curve e) were the lowest because of the poor conductivity of Nafion.

Moreover, EIS was used to further explore the electrode interface properties in terms of impedance changes. In the Nyquist plot (Figure 5B), the diameter of the semicircles was equivalent to the faradaic charge transfer resistances (R_ct_) of the modified electrodes [44]. According to the semicircles diameters, the order of the R_ct_ of different modified electrodes was obtained as follows: (BiO)_2_CO_3_-rGO-Nafion/GCE (curve e) > GO/GCE (curve b) > bare GCE (curve a) > (BiO)_2_CO_3_-rGO/GCE (curve c) > rGO/GCE (curve d). The largest semicircle can be found in the EIS plot of (BiO)_2_CO_3_-rGO-Nafion/GCE, as compared to the other modified electrodes, which indicates a slower interfacial charge transfer ability of (BiO)_2_CO_3_-rGO-Nafion/GCE because of the large interfacial resistance of Nafion. The insert is the Modified Randles circuit, representing the equivalent circuit model to fit the Nyquist curves of the EIS spectra. The analysis results of EIS are consistent with those of CV.

**Figure 6:**
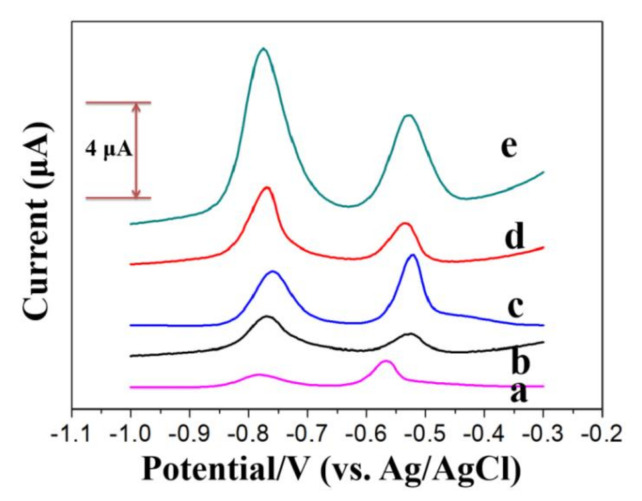
The square-wave anodic stripping voltammetry (SWASV) curves of 40 μg/L of Pb(II) and Cd(II) with bare GCE (**a**), rGO/GCE (**b**), Nafion/GCE (**c**), (BiO)_2_CO_3_-rGO/GCE (**d**), and (BiO)_2_CO_3_-rGO-Nafion/GCE (**e**).

The stripping responses of 40 μg/L Pb(II) and Cd(II) on different modified electrodes are shown in Figure 6 (bare GCE: curve a; rGO: curve b; Nafion/GCE: curve c; (BiO)_2_CO_3_-rGO/GCE: curve d; (BiO)_2_CO_3_-rGO-Nafion/GCE: curve e). The weakest stripping responses of Pb(II) and Cd(II) were obtained with bare GCE, which reflects its low sensitivity. With the rGO/GCE, there was no significant improvement, which means rGO alone does not do much to improve the sensitivity. In contrast, the much higher stripping responses of Cd(II) and Pb(II) can be seen with Nafion/GCE. Moreover, (BiO)_2_CO_3_-rGO/GCE also exhibited higher stripping peak currents then bare GCE but was not significantly higher than Nafion/GCE.

Most important of all, (BiO)_2_CO_3_-rGO-Nafion/GCE possessed not only the distinguishable and completely separated stripping peaks around −0.78 V for Cd(II) and −0.55 V for Pb(II), but also the highest stripping responses as compared with other electrodes. This revealed that (BiO)_2_CO_3_-rGO-Nafion nanocomposite has a dramatic affinity, as well as wonderful performance, toward the SWASV analysis of Cd(II) and Pb(II) because of the synergistic effect of rGO, (BiO)_2_CO_3_ and Nafion, which could be attributed to three factors. First, the presence of rGO in the composite increased the conductivity and effective specific surface area, which was beneficial to the adsorption of the probe metal ions and the transfer of redox electrons [45,46]. Second, (BiO)_2_CO_3_ efficiently helped the preconcentration of Pb(II) and Cd(II) because of the formation of multicomponent alloy with HMs, which made them reduce more easily with a higher current [25]. Third, the presence of Nafion improved the structural stability and the affinity of HMs due to its abundant sulfonate groups [47,48].

### 3.3. Analytical Performance of (BiO)_2_CO_3_-rGO-Nafion/GCE

**Figure 7:**
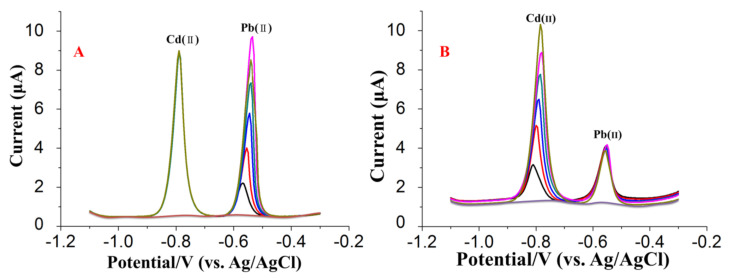
SWASV curves at (BiO)_2_CO_3_-rGO-Nafion/GCE in 0.2 M acetate buffer solution (pH 5.0) containing (**A**) different concentrations of Pb(II) in the presence of 40 μg/L Cd(II), (**B**) different concentrations of Cd(II) in the presence of 25 μg/L Pb(II).

Mutual interference could be a serious problem in the simultaneous detection of Cd(II) and Pb(II) and further practical applications. Therefore, an individual measurement of Pb(II) or Cd(II) was carried out by fixing the concentration of one of the ions, whereas the concentration of another one was changed in the meanwhile. Figure 7 shows the stripping currents of Pb(II) and Cd(II), while one’s concentration was changed and the other was kept constant. As seen in Figure 7A, in the range of 10–60 μg/L, the stripping peaks of Pb(II), in the case of keeping the concentration of Cd(II) at 40 μg/L, increased linearly with the concentration of Pb(II). Interestingly, the stripping current of Cd(II) stayed constant in the meanwhile. Similar results could be obtained by increasing the concentration of Cd(II) but keeping the concentration of Pd(II) constant at 25 μg/L, as shown in Figure 7B. This revealed that the stripping peaks of Cd(II) and Pb(II) were completely independent with each other in the binary mixtures of these ions.

**Figure 8:**
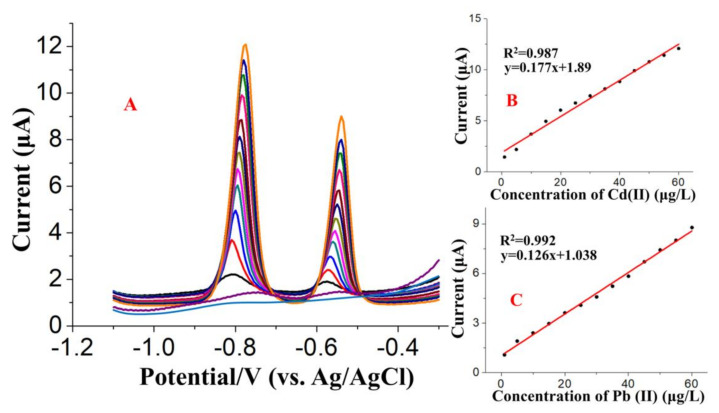
(**A**) Square wave anodic stripping voltammograms for additions of 1, 5, 10, 15, 20, 25, 30, 35, 40, 45, 50, 55 and 60 μg/L Cd(II) and Pb(II). The right part of the figure shows the calibration curves for (**B**) Cd(II) and (**C**) Pb(II).

Under the optimized experimental conditions, several SWASV were carried out to build the calibration curve of the concentration of target HMs and the stripping current using (BiO)_2_CO_3_-rGO-Nafion/GCE, and then to obtain the limit of detection and linear range. As shown in Figure 8A, the response peaks of probe Cd(II) and Pb(II) were increased linearly while increasing the concentration of these HMs in the concentration range of 1 to 100 μg/L. The calibration curves of target HMs were obtained depending on the well-defined stripping peaks observed at around −0.80 V for Cd(II) and −0.55 V for Pb(II). The corresponding fitting equations were *y* = 0.177*x* + 1.89 (*x*: μg/L, *y*: μA, 1 to 60 μg/L, r = 0.987) for Cd(II), and *y* = 0.126*x* + 1.038 (*x*: μg/L, *y*: μA, 1 to 60 μg/L, r = 0.992) for Pb(II), as shown in the Figure 8B,C. The limits of detection (LOD) were calculated as 0.24 μg/L for Pb(II) and 0.16 μg/L for Cd(II) based on the equation of LOD = 3 × StDev/Slope. The LOD is equal to the lowest amount of analyte in the sample, which can be detected but not necessarily quantitated under stated experimental conditions (USP) [49]. StDev is the standard deviation of blank response. Similar methods used for the calculation of LOD, with similar results, have been discussed in previous studies. Marinho et al. [25] developed a electrochemical sensor of a Bi_2_O_3_-modified electrode for the detection of Cd(II), Pb(II), and Zn(II), with a linear range of 1–150 µg/L and LOD of 0.26 µg/L for Cd(II), and a linear range of 2–250 µg/L and LOD of 0.52 µg/L for Pb(II), respectively. In addition, a electrochemical sensor of BiF_4_-CPE, proposed by Sopha et al. [26], exhibited favorable linearity for Cd(II) and Pb(II) in a concentration range from 20 to 100 µg/L with LODs of 9.8 and 1.2 µg/L for Cd(II) and Pb(II), respectively. Furthermore, to verify the potential of the proposed modified electrode for the determination of Cd(II) and Pb(II), several reported modified electrodes are shown in Table 1 and were further used for the detection performance comparison of different modified electrodes. According to the results presented in Table 1, the proposed (BiO)_2_CO_3_-rGO-Nafion/GCE exhibited a lower detection limit and a comparable linear range. Additionally, the (BiO)_2_CO_3_-rGO-Nafion/GCE is easy to fabricate with a low cost, and the matching instruments is also economic. In summary, the proposed modified electrode, i.e., (BiO)_2_CO_3_-rGO-Nafion/GCE, is very efficient in detection of Cd(II) and Pb(II) and also has the prospect of application in real samples.

### 3.4. Stability Verification

**Figure 9:**
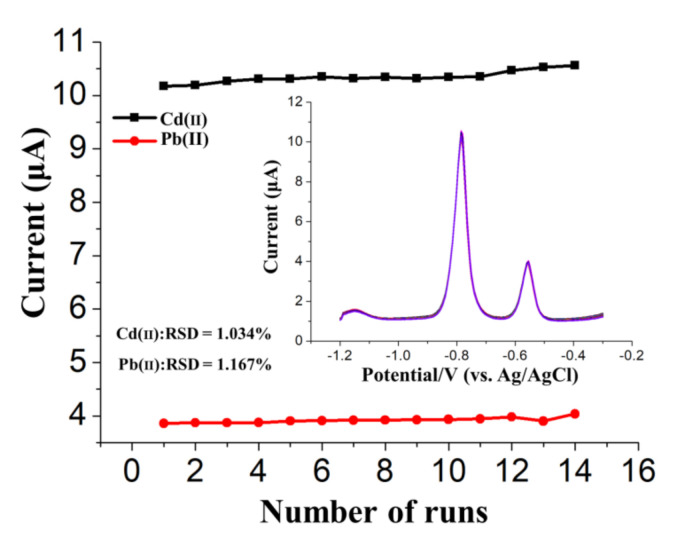
Stripping current measurements of 50 μg/L Cd(II) and 25 μg/L Pb(II) on the (BiO)_2_CO_3_-rGO-Nafion/GCE in 0.2 M acetate buffer (pH 5.0). The insets correspond to the data collected from every SWASV response for a total of six times.

In order to further evaluate the reproducibility and stability, 14 repetitive measurements of the SWASV current of 50 μg/L Cd(II) and 25 μg/L Pb(II) were carried out in a mixture of these two ions prepared using acetate buffer (0.2 M, pH 5.0). As shown in Figure 9, the stripping signals of the 14 tests showed a low relative standard deviation (RSD) of 1.034% for Cd(II) and 1.167% for Pb(II), indicating that the developed (BiO)_2_CO_3_-SWCNs-Nafion/GCE has favorable repeatability and satisfactory stability, which is attractive for further use in the analysis of trace HMs in different areas. There was an ignorable carryover of the stripping signal for the blank solution, which can be found after each measurement. However, this slight carryover did not affect the results of repeated measurements significantly.

### 3.5. Interference Studies

**Figure 10:**
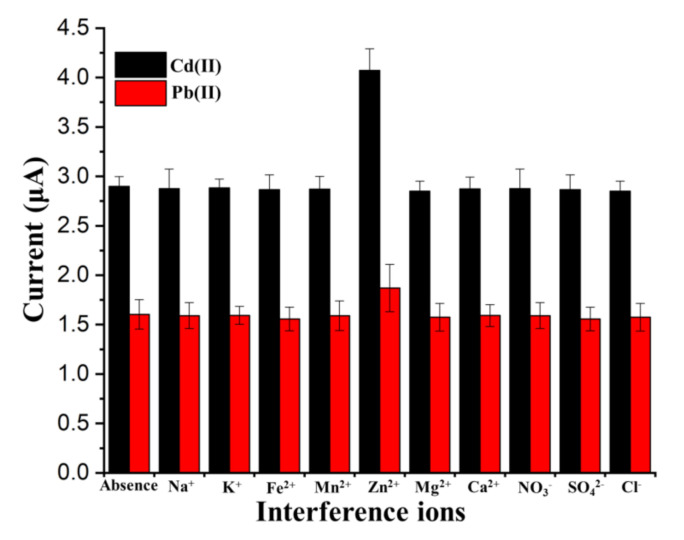
Interference study of high concentrations of different other ions on the stripping peak currents of 10 μg/L Cd(II) and Pb(II).

As shown in Figure 10, the investigation of the interference effect of nontarget metal ions with high concentrations on the stripping currents of 10 μg/L target HMs, i.e., Pb(II) and Cd(II), was performed in acetate buffer (0.2 M, pH 5.0). Under optimized conditions, after adding a 10^4^-fold level of anions, i.e., NO_3_^−^, SO_4_^2−^, and Cl^−^, the variation of the stripping responses was nonsignificant (current variation <±10%). Additionally, the metal cations of Na^+^, K^+^, Ca^2+^, Zn^2+^, Mg^2+^, Mn^2+^, and Fe^2+^ (10^4^-fold) were also used for the verification of the interference effect. The results showed that, except for Zn^2+^, no significant changes can be found in the peak current signals (current variation <±10%). In the stripping currents, increases of 40.49% and 16.58% can be found for the Cd(II) and Pb(II), respectively, in the presence of 10^4^-fold Zn^2+^. This may be because of the alloy formed between the target metals and Zn, which promoted the pre-deposition of Cd and Pb. However, the concentration of Zn^2+^ in wastewater after initial pretreatment was at trace level, which would not make a significant influence on the stripping current of Cd and Pb. Additionally, the interference of Zn^2+^ could be blocked/reduced using a smaller deposition potential, i.e., −0.9 V, which suggests that Cd(II) and Pb(II) can be electrochemically reduced onto the electrode surface under this condition.

### 3.6. Application to Synthetic Water Samples

In order to estimate the applicability of the proposed (BiO)_2_CO_3_-rGO-Nafion/GCE for the analysis of Pb(II) and Cd(II), several synthetic water samples were prepared and used for the test of the modified electrode based on SWASV. The average recoveries of Cd(II) and Pb(II) were calculated to be 100.42% and 99.98%, respectively, as shown in Table 2. Thus, it is reasonable to conclude that the (BiO)_2_CO_3_-rGO-Nafion/GCE proposed in this paper has a potential application prospect in the analysis and monitoring of Cd(II) and Pb(II) in the environmental samples.

## 4. Conclusions

In this study, a novel electrochemical sensor for the simultaneous detection of Pb(II) and Cd(II) in water samples was developed based on (BiO)_2_CO_3_-rGO-Nafion/GCE. The analytical and microscopic features of the proposed (BiO)_2_CO_3_-rGO-Nafion/GCE were characterized by SEM, EDX, FTIR, Raman, XRD, CV, EIS, and SWASV, which showed that the synergistic effect of (BiO)_2_CO_3_-rGO-Nafion resulted in a much larger specific surface area, better electrical conductivity, and higher catalytic ability than the bare electrode, and more Pb(II) and Cd(II) were electrodeposited on the surface of the electrode. The proposed (BiO)_2_CO_3_-rGO-Nafion/GCE achieved a low limit of detection, a high sensitivity, and a good stability for the SWASV analysis of Pb(II) and Cd(II). The detection results for the synthetic water samples indicated the good practicability of the proposed (BiO)_2_CO_3_-rGO-Nafion/GCE sensor, which could be potentially further applied for the analysis of Pb(II) and Cd(II) in real samples as another promising type of bismuth-based material-modified electrodes.

## Figures and Tables

**Table 1:** Comparison of different sensors used in the detection of Pb(II) and Cd(II).

Electrodes	Technique	Linear Range (μg/L)		Detection Limit (μg/L)		Reference
		Pb(II)	Cd(II)	Pb(II)	Cd(II)	
(BiO)_2_CO_3_-modified GCE	SWASV	4–150	2–125	1.15	0.65	[25]
Bi(NO_3_)_3_ salt into GEC	SWASV	20–80	10–40	11.81	7.23	[50]
Bi-powder modified CPE	SWASV	10–100	10–100	0.9	1.2	[51]
Bi_2_O_3_/SPE	SWASV	20–100	20–100	2.3	1.5	[52]
BiF_4_-CPE	SWASV	20–100	20–100	1.2	9.8	[26]
Bi-GC composite electrode	SWASV	1–100	1–100	0.41	0.49	[53]
In-situ BiSPCE	DPASV	0.83–23.3	1.35–14.5	0.25	0.4	[18]
(BiO)_2_CO_3_-rGO-Nafion/GCE	SWASV	1–60	1–60	0.16	0.24	This work

BiF_4_: Tetrafluorobismuthate, GC: Glassy carbon, BiSPCEs: Bismuth screen-printed carbon electrodes.

**Table 2:** Results of the simultaneous detection of Cd(II) and Pb(II) in synthetic water samples.

Sample No.	Found ^a^ (μg/L)		Added (μg/L)		Detected After Adding ^a^ (μg/L)		Mean Recovery (%)	
	Cd(II)	Pb(II)	Cd(II)	Pb(II)	Cd(II)	Pb(II)	Cd(II)	Pb(II)
1	4.85 ± 1.18	9.91 ± 1.12	5	10	9.79 ± 1.16	20.22 ± 1.09	98.8	103.1
2	3.96 ± 0.95	9.07 ± 1.25	10	15	14.16 ± 1.13	23.89 ± 1.12	102	98.8
3	3.12 ± 1.06	7.98 ± 1.30	15	20	18.19 ± 1.22	27.59 ± 0.98	100.47	98.05

^a^ Mean value ± Relative Standard Deviation.
